# Can anti-cyclic citrullinated peptide antibody-negative RA be subdivided into clinical subphenotypes?

**DOI:** 10.1186/ar3505

**Published:** 2011-10-27

**Authors:** Diederik PC De Rooy, Annemiek Willemze, Bart Mertens, Tom WJ Huizinga, Annette HM Van der Helm-van Mil

**Affiliations:** 1 Department of Rheumatology, Leiden University Medical Center, Leiden, The Netherlands; 2 Department of Statistics and Bioinformatics, Leiden University Medical Center, Leiden, The Netherlands

## Abstract

**Introduction:**

Studies investigating genetic risk factors for susceptibility to rheumatoid arthritis (RA) studied anti-citrullinated peptide antibody (CCP)-positive RA more frequently than anti-CCP-negative RA. One of the reasons for this is the perception that anti-CCP-negative RA may include patients that fulfilled criteria for RA but belong to a wide range of diagnoses. We aimed to evaluate the validity of this notion and explored whether clinical subphenotypes can be discerned within anti-CCP-negative RA.

**Methods:**

The 318 patients with anti-CCP-negative RA (1987 ACR criteria), included in the Leiden Early Arthritis Clinic between 1993 and 2006, were studied for baseline characteristics and radiologic progression data during a mean follow-up of 5 years. Grouping was studied both at variable and patient levels. Principal components analysis and partial least-squares regression were applied to study for clustering of variables. A cluster analysis was performed to look for clustering of patients.

**Results:**

The simultaneous presence of patient characteristics at disease presentation was observed for several groups; however, the three largest groups of patients' characteristics explained only 26.5% of the total variance. Plotting the contribution of each patient to these three groups did not reveal clustering of patients. Comparable observations were made when data on progression of joint destruction were studied in relation to baseline clinical data. A cluster analysis, evaluating whether patients resemble each other, revealed no grouping of patients. Altogether, no clinically distinguishable subphenotypes were observed.

**Conclusions:**

The current data provide evidence that, for risk-factor studies, anti-CCP-negative RA patients can be studied as one group.

## Introduction

Rheumatoid arthritis (RA) has been considered a relatively homogeneous clinical syndrome for more than 50 years. However, our current view of RA as a single disease may become untenable, and the disease may be subdivided into a range of disorders based on improved knowledge of its driving immunologic markers [1-3]. During the last decade, a number of studies suggested that RA can be divided into two syndromes: anti-citrullinated peptide antibody (CCP)-positive and anti-CCP-negative RA. This subdivision was based on differences in genetic risk factors, histopathologic differences, and differences in outcome of anti-CCP-positive and anti-CCP-negative RA [4].

Several successful genome-wide studies for genetic risk factors for anti-CCP-positive RA have been performed. Studies on genetic risk factors for anti-CCP-negative RA are thus far lacking. One of the reasons for this is the fear of phenotypic misclassification, as anti-CCP-negative RA is often considered to be a heterogeneous disease [1,5]. For future risk-factor, translational, and outcome studies on the subgroup of anti-CCP-negative RA patients, it is essential to provide epidemiologic and clinical evidence on whether anti-CCP-negative RA can be considered one entity. In this study, we therefore aimed to determine whether the group of anti-CCP-negative RA patients can be separated into clinically distinguishable subphenotypes.

## Materials and methods

### Patients

The 704 patients who were included between 1993 and 2006 in the Leiden Early Arthritis Clinic and who were diagnosed with RA according to the 1987 ACR criteria were selected; 318 patients had anti-CCP-negative RA and were therefore selected for further analysis. The Leiden Early Arthritis Clinic previously has been described extensively [6]. In short, it is a population-based inception cohort of patients presenting with arthritis to the Department of Rheumatology of the Leiden University Medical Center. This is the only referral center in a health care region of approximately 400,000 inhabitants. Written informed consent was obtained from all patients, and the cohort was approved by the local medical ethical committee (Ethics Committee of the Leiden University Medical Center). At first visit, the rheumatologist completed a questionnaire regarding the presenting symptoms, as reported by the patient: type, localization and distribution of initial joint symptoms, duration and course of the initial symptoms, and the presence of inflammatory back pain and skin abnormalities. The patient's smoking history and family history were assessed. Patients rated morning stiffness in minutes (mean, 103; SD, 112). The Health Assessment Questionnaire (HAQ) was used to provide an index of disability. A 44-joint count for swollen joints (SJC) was performed. Anti-CCP2 antibodies were measured in sera collected at baseline with enzyme-linked immunosorbent assay (ELISA) (Immunoscan RA Mark 2; Eurodiagnostica, Arnhem, The Netherlands). Samples with a value less than 25 units/ml were considered negative, according to the manufacturer's instructions. IgM-Rheumatoid Factor (RF) was determined with ELISA. RF titers ranged from 0 to 200 IU/ml. For the analyses, RF levels titers were divided into three groups: RF normal, RF moderately increased (1 to 3 times the reference value), and RF highly increased (> 3 times reference value) [7]. Anti-modified citrullinated vimentin (MCV) antibodies were also measured with ELISA (Orgentec Diagnostika, Mainz, Germany); here, the cutoff level was 20 arbitrary units, according to the manufacturer's instruction. All the mentioned baseline variables were studied here, as they may, on their own or in combination with other characteristics, point to different disease subsets. Annual radiographs of the hands and feet were taken during a mean follow-up period of 5 years (minimum, 0; maximum, 14 years) and scored according to the Sharp-van der Heijde method (SHS) by an experienced reader. The intraclass observer correlation coefficient for the radiographic progression rate was 0.97. The radiographic SHS progression per year was calculated for each patient by using a linear regression analysis with the following formula: Y = α + βx. All available radiographs per patient were used to estimate a patient's progression rate (the β in the equation). At a group level, the median SHS values (± SD) at the subsequent time points were 5.0 (9.8) at baseline; 7.0 (13.3) at 1-year follow-up; 9.0 (15.6) at 2 years; 9.0 (17.2) at 3 years; and 10.5 (22.4) at 5 years of follow-up.

### Statistical analysis

To investigate whether clinical subphenotypes can be discerned, two types of analyses were done. First, we studied whether groups of patient characteristics frequently occur together; such clustering at the variable level was studied by using the variable reduction techniques Principal Components Analysis (PCA) and Partial Least Squares regression (PLS). Second, we studied whether subgroups of patients can be discerned; such grouping at the patient level was studied by using a cluster analysis.

#### PCA

Some overlap between clinical variables is extremely common (for example, a high swollen-joint count will often be accompanied by a high number of tender joints). PCA makes use of such overlap and combines variables that frequently occur together into components. In this way, the number of variables explaining data can be reduced, which makes datasets easier to interpret. The components resulting from the PCA are based on the observed variance and not on predefined hypotheses, making this technique suitable for exploring unknown relations between variables. For each component, the loading of each variable to the component is provided. Loadings > 0.4 are generally considered relevant. For each component, an observed variance is presented, indicating the percentage of the total variance in clinical variables that is explained by this component. Here, a PCA was performed, and the contribution of each patient to the most important components was plotted to look for clustering of patients. The PCA was performed by using the following baseline variables: age at inclusion, gender, symptom duration, acuteness of the onset of symptoms (subacute, within 1 week, or insidious, over more than 1 week), morning stiffness, fatigue, fever, smoking, family history of RA, three variables on the distributions of involved joints (upper extremities, lower extremities, or both; symmetric or asymmetric; large joints, small joints, or both), C-reactive protein, RF, the presence of baseline erosions, the number of swollen joints, anti-MCV positivity, inflammatory back pain, and skin abnormalities.

#### PLS

A limitation of PCA is that it cannot take outcome measures into account. Because patient response obtained during the disease course is at least of equal importance to baseline characteristics for the aim of the present study, we also performed a Partial Least Squares regression. PLS does an analysis that is comparable to PCA, but that has the advantage that it makes use also of outcome measures [8]. Here, a PLS regression was applied to the same variables as included in the PCA as independent variables, but with the addition of the radiologic damage over time as a dependent variable. This analysis allowed us to assess whether variance between patients can be characterized by distinguishable subgroups. Also here, identified factors were plotted to look for clustering, which may represent clinical subphenotypes. The PCA and PLS analyses were done by using SPSS version 17.0 (SPSS Inc., Chicago, IL, USA). To perform PLS, the relevant SPSS extension packages were downloaded from the links provided by the official SPSS website.

#### Cluster analysis

Subsequently, a cluster analysis was performed, which, in contrast to PCA and PLS, evaluates not grouping of patient characteristics, but grouping of patients. In other words, given all characteristics that were available of the patients, this evaluated which patients resemble each other. Accordingly, not the variables denoting them, but the patients themselves are subject to combination into clusters. Hierarchic clustering was performed by using Gene Cluster, as described by Eisen *et al*. [9]

## Results

### PCA

**Figure 1:**
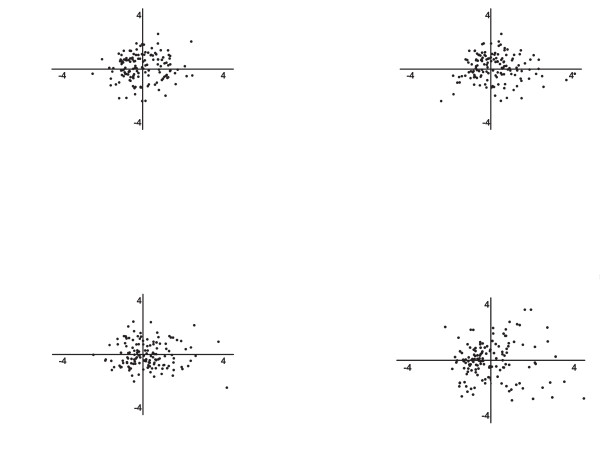
**Plots of the most important component loadings from PCA and PLS on 318 anti-CCP-negative RA patients**. In these plots, each dot indicates one single patient. Component scores indicate how strongly each component is represented in each patient. For example, in **(a)**, a dot indicates how much the variance in an individual patient is being described by factor 1 on the x-axis (age, gender, and the presence of baseline erosions) in relation to factor 2 on the y-axis (involvement of small joints versus the involvement of large joints or both SJC and CRP). If a concurrence of components was found, clustering of patients would be visible. In the PCA, clinical variables at disease onset were explored. The same applies for the factors in PLS regression. In the PLS regression, the clinical variables at disease onset were explored together with radiologic data on progression of joint destruction during a mean of 5 years of disease. CRP, C-reactive protein; PCA, principal components analysis; PLS, partial least squares regression; RA, rheumatoid arthritis; SJC, swollen joint count.

The baseline characteristics of the anti-CCP-negative RA patients are depicted in Table 1. Entering baseline variables into a PCA resulted in nine components. The first component explained 10.0% of the variance, the second component, 8.6%, and the third component, 8.0%. The relative importance of the variables contributing to the different components is depicted in Table 1. For example, in the first component, the variables age, gender, and the presence of baseline erosions were grouped. In the second component, the involvement of small joints versus the involvement of large joints or both SJC and CRP were grouped. The component scores for individual patients of factors 1 through 3 were plotted against each other, and no evident clustering was observed (Figure 1a-c). As the first three components explain most variance, these components were plotted. In these plots, each dot indicates one single patient. For example in Figure 1a, a dot indicates how much the variance in an individual patient is described by factor 1 (age, gender, and the presence of baseline erosions) in relation to factor 2 (involvement of small joints versus the involvement of large joints or both SJC and CRP).

**Table 1:** Baseline characteristics of the study population and the factors found in PCA

		Component								
Variable	Baseline frequency	1	2	3	4	5	6	7	8	9
Age at inclusion (years, mean ± SD)	59.2 (16.2)	0.761								
Female gender (*n*, %)	219 (68.9)	0.448			0.394					
Subacute onset of symptoms (versus insidious)	188 (59.1)			0.592		-0.306			0.316	
Morning stiffness (min; mean ± SD)	103.18 (112.0)				-0.496			0.484		
Fatigue (VAS; mean ± SD)	45.1 (29.9)	-0.389					0.522			
Symptom duration (days; mean ± SD)	172.1 (180.2)			0.394		-0.349			0.455	
Family history of RA (*n*, %)	62 (19.5)	-0.326		0.594		0.380				
Past or present smoking (*n*, %)	128 (40.3)				0.652			0.375		
Fever (*n*, %)	22 (6.9)							-0.319		-0.426
Involvement of small/large joints (*n*, %)	^a^		0.604							
Symmetry of involved joints (*n*, %)	216 (67.9)		-0.379		0.308	0.331				0.370
Involvement of upper/lower extremities	^b^		0.602			-0.348				
Inflammatory back pain (*n*, %)	16 (5.0)						0.466	0.531		0,321
Skin abnormalities (N, %)^c^	56 (18.1)			0.374						
Swollen joint count (mean ± SD)^d^	11.3 (8.5)		0.445	0.365						
CRP (mg/L; mean ± SD)	30.10 (34.4)	0.315	0.434							0.512
RF (IU/ml) (mean ± SD)^e^	7.12 (19.5)				0.305	-0.345	0.315		-0.602	
MCV positivity (*n*, %)	59 (18.6)			0.470	0.376		-0.321			
Erosive disease at baseline (*n*, %)	202 (63.5)	0.642					0.332			
Explained variance per component (%)		10.0	8.6	8.0	7.4	6.8	6.5	5.6	5.5	5.4

Involvement of upper extremities: shoulder, elbow, wrist, or hand joints; involvement of lower extremities: joints of hip, knee, ankle, feet or toe joints.^a^Involvement of small/large joints: in 163 patients (51.3%), only small joints of hands and feet were involved. In 53 patients (16.7%) only large joints were involved. In 93 patients (29.2%), both small and large joints were involved.^a^Involvement of upper/lower extremities: in 156 patients (49.1%) only joints in the upper extremities were involved. In 28 patients (8.8%), only joints in the lower extremities were involved. In 96 (30.2%) patients, joints in both lower and upper extremities were involved.^c^Skin abnormalities: absence or presence of dermatological abnormalities such as psoriatic lesions, ulcers, rheumatoid nodules and so on.^d^Swollen Joint Count: 44-Swollen Joint Count.^e^Analyses were performed on RF in groups (less than reference value, 1 to 3 times reference value, or more than 3 times reference value). CRP, C-reactive protein; MCV, mutated citrullinated vimentin; RF, rheumatoid factor; VAS, visual analogue scale.

### PLS

Apart from baseline characteristics, outcome data may be informative to identify differences between patient populations, so we next performed a data-reduction method that allows assessing radiologic-outcome data in addition to baseline data. To identify subsets of patients, PLS regression was used. With PLS, two latent factors were found that together accounted for 30.1% of the observed variation. The major important variables in the first latent factor were gender, symmetry of involved joints, rheumatoid factor positivity, anti-MCV positivity, age at inclusion, symptom duration at inclusion, and the presence of baseline erosions. The major variables in the second factor were the same; this suggests that little difference exists. The individual patient scores on these factors were plotted against each other. Also here, no clustering was observed (Figure 1d).

**Figure 2:**
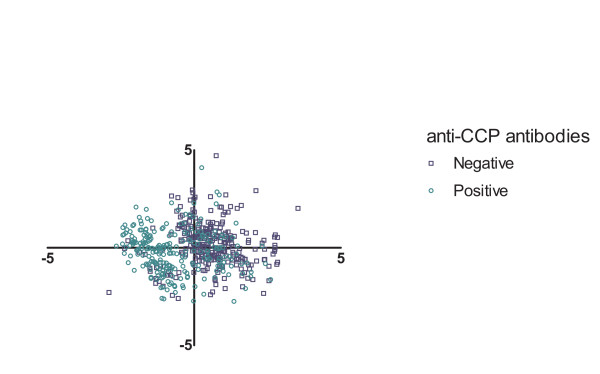
**Plots of the two major component loadings from PLS on the whole Leiden Early Arthritis Clinic (*n *= 704)**. Each dot indicates one patient. Component scores indicate how strongly each component is represented in each patient. Patients positive for anti-CCP antibodies are blue, whereas negative patients are red. CCP, citrullinated peptide antibody; PLS, partial least squares regression.

Because of the absence of clustering, we sought a positive control, to verify that the method and data used do allow finding clusters. To this end, the PLS with baseline and radiologic progression data was repeated on the total group of 704 RA patients instead of on the subgroup of anti-CCP-negative patients. Anti-CCP status was not included in this analysis, so that the analysis was not influenced by this variable. Again, two factors were found, explaining together 10.1% of the observed variance. The main variables of these components were gender, rheumatoid factor, age at inclusion, and CRP. Clustering the first two factors revealed two clusters; one for anti-CCP-positive patients and one for anti-CCP-negative patients (see Figure 2).

### Cluster analysis

**Figure 3:**
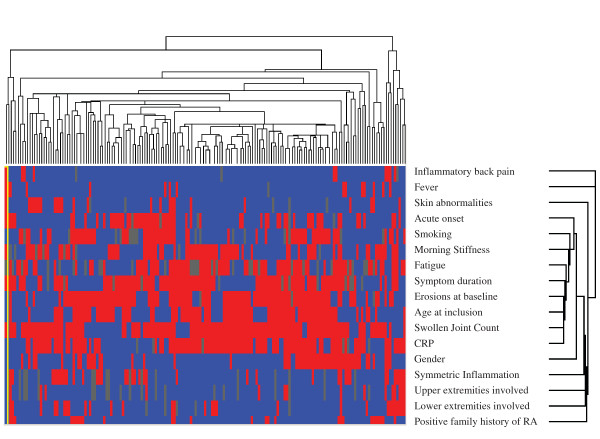
**Heat map of the cluster analysis of 318 anti-CCP-negative RA patients**. Heat map representing the presence or absence of disease characteristics in individual patients. To make variables comparable, all values were transformed into binary values. For those variables on a continuous scale, the following cut-offs were made: Morning Stiffness, ≤60 minutes; Fatigue, fatigue rated more than mean (45.1) on Visual Analogue Scale; symptom duration more than or ≤12 weeks age at inclusion, age greater than mean age (59.2 years); swollen joint count, more than four swollen joints; CRP, CRP greater than reference value (10 mg/L). The dendrograms depict the relative strength of correlations between the variables and the patients, respectively. CCP, citrullinated peptide antibody; CRP, C-reactive protein; RA, rheumatoid arthritis; SJC, swollen joint count; VAS, visual analogue scale.

Finally, we explored whether anti-CCP-negative patients can be grouped into subgroups of patients with similar characteristics. To this end, a heat map was made in which the patients with the most similarity clustered together. Cluster analysis showed no clustering of patients in the heat map, and this finding is supported by the dendrogram (Figure 3). Therefore, also with these analyses, no distinguishable groups of patients with similar characteristics were recognized.

### Sensitivity analysis

Patients negative for anti-CCP antibodies can harbor other autoantibodies. Here 24.2% of the anti-CCP-negative patients were positive for RF, and 18.6% were positive for anti-MCV. It can be argued that it is more appropriate to perform the analyses on patients negative for anti-CCP, RF, and anti-MCV. Therefore, all analyses were repeated in this subgroup (*n *= 171). Similar observations were made (data not shown).

## Discussion

This study determined whether anti-CCP-negative patients fulfilling the 1987 ACR criteria for RA can be subdivided into clinical subphenotypes and explored extensive phenotypic characteristics at baseline, as well as data on progression in joint destruction during the disease course. In addition, several methods were applied, intending to find subgroups of either variables or patients. With any method used, no clearly distinguishable clusters were observed, although the methods used did distinguish CCP-positive and CCP-negative patients as identifiable subphenotypes. Therefore, these data do not support the hypothesis that anti-CCP-negative RA is composed of different subsets, but rather provide evidence that anti-CCP-negative RA can be regarded as one disease, and therefore, risk-factor studies in anti-CCP-negative RA are feasible.

The data evaluated concerned a wide variety of clinical characteristics, such as the acuteness of the onset of symptoms, the distribution of involved joints, the severity of fatigue, fever, skin abnormalities, inflammatory back pain, acute-phase reactants, and radiologic baseline and progression data. The variables assessed are, in our view, variables that might in combination form patterns characteristic of different disease subsets. However, despite the evaluation of a large range of characteristics at disease presentation and the evaluation of long-term radiologic follow-up data, no clear subphenotypes were discerned. We cannot exclude that when other variables are assessed, conclusions might be different.

To test whether the currently used data and methods are able to find clinical subsets, we also studied the total RA population, including 704 patients, of whom 318 were anti-CCP-negative patients. We observed that PLS regression is able to discriminate between anti-CCP-positive and anti-CCP-negative disease. This is in line with published data that anti-CCP-positive RA has more progressive joint destruction during the disease course than does anti-CCP-negative RA [10]

The present study did not aim to find statistically significant associations of baseline variables with the outcome. The present study also does not give any indication on whether the pathogenesis of anti-CCP-negative RA is heterogeneous or homogeneous between patients. We explored whether clinical data provide evidence that different groups of patients compose the group of anti-CCP-negative RA patients. If subclinical phenotypes had been identified, this is relevant for future pathophysiological studies. Then it could be suggested that, to prevent phenotypic misclassification, studies should be done on anti-CCP-negative subphenotypes. The present observation of a lack of phenotypic heterogeneity within anti-CCP-negative RA suggests that future studies on pathogenic mechanisms underlying anti-CCP-negative RA can be done on the total population of anti-CCP-negative patients.

## Conclusions

Based on the present data, we suggest that risk factors studied on the anti-CCP-negative RA patients can be performed on the total group of 1987 ACR criteria-positive, anti-CCP-negative RA patients.

## Abbreviations

CCP: citrullinated peptide antibody; CRP: C-reactive protein; HAQ: Health Assessment Questionnaire; MCV: modified citrullinated vimentin; PCA: principal components analysis; PLS: partial least-squares regression; RA: rheumatoid arthritis; RF: rheumatoid factor; SHS: Sharp-van der Heijde Score; SJC: swollen joint count; VAS: visual analogue scale.

## Competing interests

The authors declare that they have no competing interests.

## Authors' contributions

DR performed the statistical analyses and wrote the first version of the manuscript. AW and BM assisted with the statistical analyses. AH and TH were responsible for the selection of patients. All authors contributed to revising and adjusting the manuscript. All authors have read and approved the manuscript for publication.
